# Cancer Patients Have a Higher Risk Regarding COVID-19–and Vice Versa?

**DOI:** 10.3390/ph13070143

**Published:** 2020-07-06

**Authors:** Franz Geisslinger, Angelika M. Vollmar, Karin Bartel

**Affiliations:** Pharmaceutical Biology, Department Pharmacy, Ludwig-Maximilians-University of Munich, 81377 Munich, Germany; franz.geisslinger@cup.uni-muenchen.de (F.G.); angelika.vollmar@cup.uni-muenchen.de (A.M.V.)

**Keywords:** SARS-CoV-2, COVID-19, cancer, inflammation, oncovirus

## Abstract

The world is currently suffering from a pandemic which has claimed the lives of over 230,000 people to date. The responsible virus is called severe acute respiratory syndrome coronavirus 2 (SARS-CoV-2) and causes the coronavirus disease 2019 (COVID-19), which is mainly characterized by fever, cough and shortness of breath. In severe cases, the disease can lead to respiratory distress syndrome and septic shock, which are mostly fatal for the patient. The severity of disease progression was hypothesized to be related to an overshooting immune response and was correlated with age and comorbidities, including cancer. A lot of research has lately been focused on the pathogenesis and acute consequences of COVID-19. However, the possibility of long-term consequences caused by viral infections which has been shown for other viruses are not to be neglected. In this regard, this opinion discusses the interplay of SARS-CoV-2 infection and cancer with special focus on the inflammatory immune response and tissue damage caused by infection. We summarize the available literature on COVID-19 suggesting an increased risk for severe disease progression in cancer patients, and we discuss the possibility that SARS-CoV-2 could contribute to cancer development. We offer lines of thought to provide ideas for urgently needed studies on the potential long-term effects of SARS-CoV-2 infection.

## 1. Introduction

Over the past few months, the novel coronavirus, called severe acute respiratory syndrome coronavirus 2 (SARS-CoV-2), has become a worldwide concern, globally threatening public health. Originating in Wuhan, China, it dramatically spread to countries all over the world, causing a pandemic [1]. As of the 3rd of May 2020, there are more than 3.3 million people infected with the novel coronavirus SARS-CoV-2 and more than 230,000 people have died from the associated disease, called coronavirus disease 2019 (COVID-19) [2].

SARS-CoV-2 belongs to the subfamily of betacoronaviruses and is an enveloped ssRNA virus. Like most other coronaviruses, SARS-CoV-2 is a zoonotic virus, suggested to have its origin in bats, from which it was transmitted to humans in December 2019 [3,4]. In comparison to earlier identified SARS-related coronaviruses, namely SARS-CoV-1 and MERS-CoV, SARS-CoV-2 is more contagious and, like SARS-CoV-1, SARS-CoV-2 is also transmitted from human to human [3]. Subsequently, there are more infections and fatalities documented for SARS-CoV-2 as compared to SARS-CoV-1 [2,5], despite a supposedly lower lethality [6]. As it is a novel identified virus, there is no vaccine available to prevent SARS-CoV-2 infection and moreover there are no licensed drugs available for therapy [7,8].

The main symptoms of COVID-19, the lung disease following SARS-CoV-2 infection are fever, cough, shortness of breath and respiratory distress syndrome with risk for septic shock. Furthermore, lymphopenia, myalgia, nausea and vomiting frequently occur [9,10]. Interestingly, about 80% of infected people have no or only mild symptoms, while only approximately 20% have severe events, making hospital stays and ICU support necessary [11]. The reason behind this phenomenon remains predominantly unknown, but it is hypothesized that the outcome is linked to overshooting immune responses [12]. Furthermore, severity seems to correlate with age and comorbidities, as older people as well as patients with comorbidities, including cancer, have a higher risk for severe events and a poor outcome [13,14]. Recent studies have mainly focused on acute complications upon SARS-CoV-2 infection. However, viral infections may also have long-term consequences in terms of risk for cancer development, mainly mediated by inflammatory tissue damage [15].

**Figure 1:**
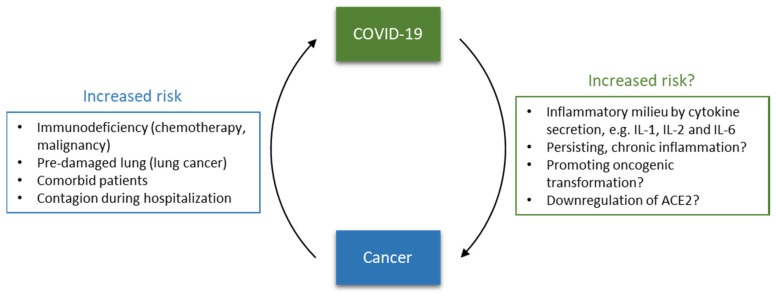
Bidirectional relationship between coronavirus disease 2019 (COVID-19) and cancer.

In this context, this opinion focuses on the interplay between cancer and SARS-CoV-2, summarizing the risk of cancer patients for SARS-CoV-2 infection and COVID-19 fatality. Furthermore, the immune system and inflammation are put into focus as significant factors of fatality. Additionally, potential capability and mechanisms of SARS-CoV-2 to contribute to cancer development are discussed, opening questions to be addressed in future research (Figure 1).

## 2. Increased Risk for Cancer Patients?

As already mentioned above, age and comorbidities are the main factors for severe COVID-19 progress and fatality, putting patients at increased risk [16,17,18]. These comorbidities are very versatile, including hypertension, cardiovascular diseases, diabetes, chronic obstructive pulmonary disease (COPD), and diseases [13,14]. Initial studies suggested that all cancer patients, regardless of the affected organ or tissue, are more at risk regarding a severe COVID-19 progression and, subsequently, fatality. There is evidence that cancer patients are around 5 times more at risk of dying from COVID-19 than patients without comorbidities [17,18]. In contrast, Dai et al. and Kong et al. recently pointed out that only patients suffering from hematological, lung or metastatic cancer have an increased risk for severity and fatality as compared to healthy people [19,20]. Due to the recentness of the SARS-CoV-2 pandemic, these studies were only based on small patient cohorts. Therefore, further investigations are necessary to assess the susceptibility of cancer patients regarding COVID-19 fatality for certainty.

However, increased risk for fatalities of cancer patients is not restricted to SARS-CoV-2 infections, but occurs upon various infectious diseases, such as influenza, varicella-zoster, tuberculosis and infections with *Toxoplasma gondii* [21,22,23,24]. The supposedly higher fatality rates of cancer patients might be caused by their susceptible immune system. Anti-cancer therapy, including chemotherapy and radiotherapy, but also malignancy itself, contribute to immune suppression. In line with that, patients who received chemotherapy up to 14 days prior to infection have a much higher risk for a severe outcome than other cancer patients [17,18,25]

Therefore, oncologists recommend isolating cancer patients and testing them for SARS-CoV-2 infection regularly to protect this vulnerable group to the best ability from infection and thus severe COVID-19 progression and fatal events [26,27]. Given the high risk for fatalities, especially for cancer patients receiving chemotherapy, the resulting altered risk benefit ratio during the SARS-CoV-2 pandemic should be taken into consideration.

## 3. Inflammatory Response in COVID-19

For a proper evaluation of cancer and COVID-19 relation and vice versa, a detailed understanding of the underlying pathogenesis is mandatory.

While SARS-CoV-2 infection predominantly affects the respiratory tract, developing COVID-19, resulting in fever, cough, shortness of breath and respiratory distress syndrome [9,10], this might not be the major reason for causing severe events. Interestingly, an overshooting immune response resulting in a cytokine storm and the modulation of angiotensin converting enzyme 2 (ACE2) have been connected to severity.

### 3.1. Cytokine Storm As Major Complication

A cytokine storm or cytokine releasing syndrome is described as a quick and massive cytokine release which may lead to multiple organ failure. High amounts of pro-inflammatory cytokines are secreted after the activation of immune cells, initiating an overshooting immune response [28]. The subsequent, severe inflammation can cause acute, life-threatening disorders by inducing septic shock, impairing proper oxygen absorption, inducing lung failure and multi-organ failure [29,30,31,32]. Cytokine storms frequently occur as complications after infection with pathogens, such as SARS-CoV-1, Epstein-Barr virus and influenza virus, for example, as well as a side effect of cancer therapy [33]. Preliminary evidence suggests that such a cytokine storm in response to infection with SARS-CoV-2 is a major factor, promoting severe COVID-19 progress and subsequently disease fatality [8,12].

In detail, it has been reported by Wan et al. that in a study with patients that suffered from severe COVID-19 pneumonia (21 patients included), levels of CD8^+^ T-cells and B-cells were reduced, while the cytokines IL-6 and IL-10 were elevated as compared to patients with a mild pneumonia (102 patients included) [34]. These findings were confirmed by other studies, showing elevated levels of pro-inflammatory cytokines of COVID-19 patients. In this context, a study of Qin et al. on 452 COVID-19 patients, of which 286 were classified as severe cases, revealed significantly elevated levels of IL-2, IL-6, IL-8, IL-10 and TNFα associated with the disease [35]. IL-2, IL-6, and IL-10 were furthermore found to be remarkably upregulated in patients with severe COVID-19 in a study by Shi et al. [36]. Along this line, Huang et al. reported increased plasma levels of pro-inflammatory mediators IL-2, IL-7, IL-10, GSCF, IP10, MCP-1, MIP-1A, and TNF-α in COVID-19 patients that were hospitalized in an ICU. However, this study only included 49 patients [6]. Consequently, mild and severe cases seem to show a different cytokine secretion profile, providing an option as biomarkers to monitor disease progression.

On the other hand, targeting excessive cytokine release with immunosuppressive agents might serve as a therapeutic option for severe SARS-CoV-2 infection [12]. Evidence suggests that patients suffering from rheumatism which are typically treated with cytokine blockers, such as infliximab, adalimumab and ustekinumab, do not have a higher risk for severe COVID-19 progress, despite their comorbidity [37]. Subsequently, cytokine blockers, especially the IL-6 antagonist tocilizumab, are considered as potential drugs to prevent cytokine releasing syndrome [38]. However, a major problem of an immunosuppressive therapy approach is optimal timing, as, especially in the initial phase, a properly working immune system is of great importance [39]. In this context, monitoring of cytokine levels in bronchoalveolar lavage fluid and peripheral blood mononuclear cells as well as virus load could be a suitable approach. [40]

### 3.2. The Role of ACE2

In addition to cytokine release syndrome, fatality has recently been connected to the modulation of ACE2. ACE2 is physiologically responsible for the conversion of angiotensin II to angiotensin 1–7 and is therefore part of the renin-angiotensin system, playing an important role in the homeostasis of the cardiovascular and renal system (reviewed in [41,42]). In addition, angiotensin II and angiotensin 1–7 also play an important role in inflammation. Angiotensin II promotes inflammation and vasoconstriction by activating AT1 and AT2 receptors, while angiotensin 1–7 has anti-inflammatory properties and, moreover, causes vasodilatation [43,44,45]. Furthermore, it is widely known that ACE2 is important for lung function, and accumulation of angiotensin II is a marker for pulmonary arterial hypertension disease progression [41]. Subsequently, ACE2 dysregulation could promote overshooting immune responses.

Intriguingly, SARS-CoV-2 invades cells via ACE2, as it was shown that its spike proteins bind to ACE2 and that ACE2 is necessary for virus entry [46,47]. After virus entry, ACE2 is probably downregulated, causing accumulation of antiogensin II and thereby influencing immune response [48,49]. As ACE2 is also expressed in the cardiovascular system, it is likely that SARS-CoV-2 might also cause cardiovascular complications. Indeed, it has been reported that a proportion of COVID-19 patients suffer from myocardial damage and heart failure [50,51]. Interestingly, these patients complain only about cardiovascular symptoms like heart palpitations and chest tightness, but not about common respiratory symptoms connected to COVID-19 [51].

Of note, studies showed that SARS-CoV-1, whose spike protein has a high homology to SARS-CoV-2 [48], binds to ACE2 and modulates its expression during SARS disease [52,53]. Interestingly, coronaviruses causing common colds, such as NL63, which also enters its host cells via ACE2, does not influence ACE2 expression [52]. Therefore, ACE2 modulation is considered as a major factor for severity, probably mediated by dysregulation of the immune response.

## 4. Inflammation Causing Cancer

Inflammation, in principle, is an essential physiological process, which involves the activation, recruitment, and action of cells belonging to the innate and adaptive immunity and protects the body from harmful pathogens. Yet, when deregulated or persisting, inflammation can cause severe diseases, such as diabetes, atherosclerosis and rheumatoid arthritis [54,55]. Usually, acute inflammation is resolved quickly with the removal of the pathogen from the body; however, if the pathogen cannot be removed completely, the inflammatory response becomes chronic [56].

An estimated 15–20% of cancers are preceded by inflammation within the same tissue, that is initiated long before tumor formation. Causes of these chronic inflammations can be pathogenic infections, such as hepatitis virus or helicobacter pylori infections, autoimmunity, or environmental factors, like alcohol or cigarette smoke [55]. The major problem with unresolved, persistent inflammation is the generation of a pro-tumorigenic environment by immune cells and their secreted cytokine mediators. This microenvironment is rich in reactive oxygen species (ROS), such as superoxide, nitric oxide, hydrogen peroxide, or radicals. Continuous exposition to ROS kills infected cells, yet also poses a threat to healthy host cells, as it drives DNA mutations [57]. Furthermore, immune cells infiltrate the site of inflammation and secrete cytokines like TNF-α, TGF-β, IL-1β and IL-6, which increase vascular permeability and favor a mesenchymal phenotype of cells that is important for migration. Along this line, matrix metalloproteinases are also secreted to digest the extracellular matrix proteins and enable migration (reviewed in [56]).

As described above, there are some studies that investigated cytokine levels in patients suffering from COVID-19 and revealed an elevation in IL-2, IL-6, IL-8, IL-7, IL-10, G-CSF, IP10, MCP-1, MIP1A, and TNF-α [6,34,35,36]. These data indicate that SARS-CoV-2 infection might bear the potential to cause carcinogenic inflammations. Of note, elevated levels of MCP-1, TGF-β1, TNF-α, IL-1β, and IL-6 were found in patients that died from the related SARS-CoV-1 infection [58]. However, whether SARS-CoV-2-induced inflammation can become chronic is unknown to date and should be a part of further research.

If chronic, the damage to healthy tissue caused by inflammatory processes is often followed by excessive tissue remodeling, leading to fibrosis. Inflammation-induced fibrosis is accompanied by a loss of organ function and tumorigenesis, for instance in the development of hepatocellular carcinoma and lung cancer [59,60]. In hepatocellular carcinoma, chronic inflammation, especially that mediated by ROS, induces the proliferation of hepatocytes, and activates hepatic stellate cells and myofibroblasts, which create extracellular matrix proteins. These are key steps that lead to fibrosis, accompanied by the genomic instability of constantly proliferating hepatocytes and alterations in blood flow. Subsequent production of VEGF drives neoangiogenesis and promotes tumor survival [61]. Similar mechanisms are described in the lung. Lung fibrosis can be driven by TGF-β, which induces myofibroblasts and the synthesis of ECM proteins and promotes tumorigenesis [62]. Again, specific data on SARS-CoV-2 infection in that regard are scarce, yet there is a study which reports pathological changes to the lung. Zhang et al. reported a case study of a 72-year-old man who suffered from COVID-19, had a rapid progression of the disease which required ICU support, and later died from the disease. The postmortem transthoracic needle biopsies showed pathologic changes in the lung displayed by diffuse alveolar damage with loose fibrous plugs [63]. Related to that, a study of seven patients that died from SARS-CoV-1 infection revealed diffuse alveolar damage, with the presence of multinucleated pneumocytes. Additionally, they discovered mild to moderate fibrosis in the lung [64]. Besides these first hints, which connect SARS-CoV infections with fibrosis, the fact that mechanical injury to the lung by intubation and high pressure ventilatory assistance in ICU support are also capable of inducing fibrosis should not be neglected [65]. Hence, infections that cause excessive and persisting inflammation and pathogenic conditions favoring fibrotic lesions bear the risk of developing tumors over time.

While it is not clear to date whether infection with SARS-CoV-2 leads to persisting pathogen presence or continuous low-level inflammatory processes, there are few in vitro studies on the related SARS-CoV-1 viral infection and cellular persistence. Chan et al. analyzed seven different cell lines for their permissiveness to SARS-CoV-1 infection. They found that the cell line permissive for infection also showed a persistent chronic infection, which could also be maintained during passaging [66]. Their findings are supported by those of Palacios et al., who reported the presence of viral particles in infected cells after multiple passaging [67]. Furthermore, Pacciarini et al. reported that SARS CoV-1 infection persisted in proximal tubular epithelial cells but not in glomerular mesangial cells. These findings indicate that the virus may cause persistent infection and that this might be cell type-dependent and yet not restricted to the lung [68]. In summary, these studies point to the possibility that SARS-CoV-2 might also be able to form persisting infection sites. However, caution is necessary when interpreting these studies, as of course transferring in vitro findings to in vivo is difficult. Interestingly, a study by Ling et al. analyzed the clearance of viral RNA in oropharyngeal swabs and feces and reports that the median time from onset of the symptoms to clearance is 9.5 and 11 days, respectively. They further found that the time until tests turned negative was delayed by glucocorticoid therapy [69]. As the time until clearance from oropharyngeal swabs and feces differs, it is not clear how the virus is transported in the body and whether negative samples are proof for the eradication of the virus. Hence, presently it cannot be estimated if SARS-CoV-2 is able to form persistent infection and studies addressing this question are urgently needed.

Despite the drawbacks of existing studies on SARS-CoV-2 and the limited information on related SARS-CoV-1 infection, these studies nevertheless indicate a pro-inflammatory phenotype, that might favor carcinogenesis, especially if it turns out to be persistent. In this regard, we suggest that follow-up studies on a broad number of COVID-19 survivors would be beneficial to assess the risk of possible long-term consequences of the disease.

## 5. Viral Infections as Major Risk Factor for Cancer—What about SARS-CoV-2?

### 5.1. Characteristics of Oncoviruses

The idea that viral infection might be the reason for the development of cancer is not new. In the 1980s, researchers hypothesized that viral infection with the human papilloma virus (HPV) could lead to the development of genital cancer [70]. This hypothesis was later proven and even rewarded with a Nobel prize for physiology and medicine in 2008 [71]. Of course, since then a lot of research has been done in the field. Viruses that are capable of causing tumorigenesis are termed oncoviruses and are currently estimated to be responsible for about 15–20% of cancerous diseases [59,72]. To date, several viruses have been shown to cause cancer by influencing a various number of cancer cell hallmarks that have been described by Hanahan and Weinberg [73], among them human papilloma virus (HPV), hepatitis virus B and C (HBV, HCV) or the Epstein-Barr virus (EBV) [59,72,74]. In addition, several other viruses have been implicated in carcinogenesis [75] (Table 1).

However, the necessary prerequisites and mechanisms that lead to cancer development upon oncovirus infection are still not entirely clear. Nevertheless, several characteristics are common among oncoviruses. They are widespread among the population, and also in individuals that do not develop cancer, they seem to cause persistent infection rather than leading to host cell lysis, and they are usually not sufficient to cause cancer on their own, but need an additional risk factor (e.g., inflammation, co-infection, immune suppression or host mutations) [59,91]. In general, oncoviruses can be classified in direct oncoviruses like EBV, HPV, HTLV-1, KSHV or MCPyV, which activate oncoproteins, either encoded by the virus or the host cell, or indirect oncoviruses. Indirect oncoviruses mainly cause chronic inflammation processes, that will eventually lead to tumor development, a mechanism typical for HBV and HCV [59]. Usually, virus-driven cancers take about 15–40 years post-infection to develop. During this period, virus replication is either strongly diminished or even absent and the virus only exists in form of its nucleic acid, present as episome, plasmid or integrated into the host genome [72,92]. Infection with the HCV virus, for example, causes carcinogenesis over a period of 20–40 years by direct and indirect oncogenic events. Direct events include interactions with host factors leading to alterations in transcriptional regulation, metabolism and apoptosis [93]. Indirect events are oxidative stress and inflammation, the driving factors of HCV oncogenesis [94].

### 5.2. Oncovirus Induced Inflammation

Even though oncoviruses are roughly categorized in direct and indirect oncoviruses, this does not mean that the tumorigenic mechanisms are exclusive. As a matter of fact, many oncoviruses are associated with inflammation. Of course, infection with the indirect oncovirus HCV causes the release of pro-fibrinogenic factors, such as TGFβ, and a chronic low-level inflammation caused by the IFN-γ- and IL-2-dominated immune response. The cytokine milieu and related production of reactive oxygen species is a strong driving factor for genetic mutations and hence cancer development [94,95].

Nevertheless, direct oncoviruses, such as EBV, HPV or KSHV, have also been linked with inflammatory immune responses. In the case of EBV, the viral protein LMP-1 activates signaling via the PI3K/Akt, the JAK/STAT and the NFκB pathway induces cancer cell hallmarks and, in that regard, tumor-promoting inflammation [96,97,98]. Furthermore, EBERs, viral RNA transcripts, activate the IL6/STAT3 pathway and induce IL6, IL9, IL10 and IGF-1, which promote inflammation and growth [99,100]. The release of inflammatory cytokines IL6, IL8, TNFα, MIP-1α and MIP-1β has also been reported following KSHV infections and is linked to the viral protein kaposin B [101,102,103]. Along this line, the expression of HPV viral protein E6 is linked to alterations in cytokine levels [104,105]. E6 facilitates the activation of NFκB, IL6 production and subsequent autocrine and paracrine activation of STAT3 [106].

Promoting inflammation is therefore a common feature of oncoviruses, and the IL6/STAT3 axis is frequently activated. IL6 is a pleiotropic, inflammatory mediator, which can activate MAPK or PI3K signaling and STAT3. This blocks apoptosis and favors cell proliferation, even in a harsh, inflammatory environment, thereby essentially contributing to malignant transformation [107].

### 5.3. What about SARS-CoV-2?

The recent out-break of SARS-CoV-2 with a rising number of infected individuals brings about the question about possible long-term consequences, such as a potential risk of developing cancer after SARS-CoV-2 infection. Since the virus has only recently emerged as a human infection, there are no studies available so far. However, there are some hints that might indicate pro-tumorigenic activity of SARS-CoV-2 which we want to point out in the following paragraph to provide ideas for future research in this area.

As described above, increased cytokine levels, including those of IL-6, have also been reported in COVID-19 patients. Whether the elevation of IL-6, which is typical for oncoviruses, is a feature of SARS-CoV-2 infection or is an unspecific consequence of the cytokine storm in these patients remains unknown to date. However, we suggest that the potential of SARS-CoV-2 to elevate cytokines, especially IL-6, and the subsequent activation of pro-inflammatory signaling pathways should be addressed in future studies, helping to estimate an oncogenic potential of SARS-CoV-2

As mentioned, there are no studies on SARS-CoV-2 in that regard; however, there are a few publications that investigated the relation between SARS-CoV, the cause of the SARS disease and now known as SARS-CoV-1, and cancer incidence. These studies might be able to guide future research on the relation of cancer and SARS-CoV-2. For instance, Li et al. reported a study on a connection between the SARS disease and childhood acute lymphatic leukemia (ALL) by the Hong Kong Paediatric Haematology and Oncology Study Group. They analyzed the development of novel childhood ALL cases prior to, during and after 2003, the year in which Hong Kong suffered from a SARS pandemic. The study shows a decline in standard-risk ALL incidence during the time of social isolation measures that were taken to prevent SARS-CoV-1 from spreading. While the study links this decrease to a less likely infection with SARS-CoV-1, the authors could not rule out other infections as a cause and only concluded that the reduced number of infections in general is responsible [108]. Another study reported an interaction of the endoribonuclease Nsp15, which is commonly expressed in coronaviruses, with the tumor suppressor protein retinoblastoma (pRb). The authors showed that Nsp15 could bind to pRb, leading to a reduction in pRb levels and alterations in its regulation of cell growth and gene expression [109]. Of note, established oncoviruses like HCV, EBV, KSHV, MCPyV, HPV, HBV, HTLV, are also known to interact with pRb [72]. EBV protein EBNA-3, KSHV protein LANA1, MCPyV or HCV protein NS5B, lead to the inactivation and degradation of Rb, which leads to an enhancement in cell cycle progression [95,102,110,111,112,113,114,115]. Therefore, the ability to modulate Rb function seems to be a hint for oncogenic potential. Studies which investigate the ability of SARS-CoV-2 to modulate tumor suppressor function, in particular Rb function, would shed interesting light on this matter.

Along this line, SARS-CoV-1 was also shown to interfere with a number of signaling pathways, that are associated with the malignant transformation of cells, such as p53, EGFR, JAK/STAT, or MAPK signaling [116]. For instance, the virus directly interacts with the protein RCHY1, an E3 ubiquitin-ligase, which leads to enhanced degradation of the tumor suppressor p53 [117]. Furthermore, SARS-CoV-1 activates p38 MAPK and subsequently elevates pro-inflammatory cytokines, including IL6 [118,119]. Interestingly, EGFR signaling is overactivated after viral infection and linked to the development of fibrosis [120,121]. As discussed above, fibrosis is highly linked to cancer development, giving further hints that SARS-CoV-1 might favor tumorigenesis. If, however, this is the case, and to what extent these pathology mechanisms also apply to SARS-CoV-2, is unknown as of yet. Nevertheless, we suggest that future research should investigate the discussed pathways after SARS-CoV-2 infection to shed light on the matter.

Interestingly, the angiotensin-converting enzyme 2/angiotensin-(1–7)/mitochondrial assembly receptor (ACE2/Ang-(1–7)/MasR) axis is debated for its role in cancer. There have been studies suggesting the pro- and anti-tumorigenic potential of its activation (reviewed in [4]). As mentioned above, the SARS-CoV-2 virus uses ACE2 to invade cells, probably followed by the downregulation of the receptor [48,52,53]. This deregulation in ACE2 could therefore favor carcinogenesis.

These studies suggest that infection with coronaviruses such as SARS CoV-1 and SARS-CoV-2 might influence carcinogenic events. It would therefore be important in our opinion to dedicate future research to address this question.

## 6. Conclusions

The novel coronavirus SARS-CoV-2, which causes COVID-19, has recently emerged as major worldwide health problem, driving health care systems to their limits and causing many deaths, especially in high-risk patient cohorts. High risk factors for a severe progression are high age and comorbidities, especially cancer. Chemotherapy- and radiation therapy-induced immunosuppression is a major risk factor for cancer patients to acquire a severe and probably fatal SARS-CoV-2 infection. Hence, SARS-CoV-2 and cancer form a dangerous couple.

However, this connection might be of a bidirectional nature, as there are some initial hints, suggesting that an infection with SARS-CoV-2 might lead to long-term pathological consequences, such as cancer development. Of course, since the virus has just recently emerged, there are no specific data available so far; however, the pathogenesis of COVID-19 shows parallels to other oncovirus infections. The excessive inflammatory immune response during severe COVID-19 cases might drive cancer progression. Furthermore, inflammatory processes, as well as mechanical lung injury potentially caused by ventilatory assistance during ICU stay, can lead to fibrosis. Inflammatory and fibrotic changes caused by viral infection are known to be cancer drivers. Therefore, studies specifically designed to investigate a possible tumorigenic potential of SARS-CoV-2 are urgently needed. Currently, a lot of strategies are being discussed concerning management of SARS-CoV-2 infections. While the world is waiting for the first vaccines to be approved, it is also hypothesized to induce herd immunity by gradually infecting most of the population with the virus. However, in this regard, caution is necessary, as the long-term consequences of infections, also with an initial mild or symptom-free progression, are not clear.

Overall, further research is greatly needed to understand SARS-CoV-2 pathogenesis and to develop vaccines and therapies. It is essential to keep in mind that the currently available studies on SARS-CoV-2 include only small numbers of patients and they therefore have to be interpreted with caution and the statistical resilience has to be proven by studies including large cohorts. In this regard, we think that follow-up studies on a broad number of COVID-19 survivors are inevitable to assess the risk of possible long-term consequences of the disease, especially regarding oncogenic potential.

## Figures and Tables

**Table 1:** Oncoviruses and respective Cancers.

Virus	Cancer	Publication
**EBV**	Burkitt’s lymphoma, Non-Hodgkin lymphoma, posttransplant lymphoproliferative disorder, nasopharyngeal carcinoma	[76,77]
**HPV**	Cervical carcinoma, Head and neck cancer	[78,79]
**HBV**	Hepatocellular Carcinoma	[80]
**HCV**	Hepatocellular Carcinoma	[81]
**HTLV-1**	Adult T-cell leukemia	[82,83,84]
**KSHV**	Kaposi’s sarcoma	[85]
**MCPyV**	Merkel cell carcinoma	[86,87,88]
**HCMV**	Mucoepidermoid carcinoma	[89,90]

EBV (Epstein-Barr virus), HPV (human papilloma virus), HBV (hepatitis virus B), HCV (hepatitis virus C), HTLV-1 (Human T-cell Lymphotropic Virus), KSHV (Kaposi’s Sarcoma Herpesvirus), MCPyV (Merkel Cell Polyomavirus), HCMV (human cytomegalovirus).
